# Fulminating Mannite

**Published:** 1847-10

**Authors:** 


*Fulminating Mannite.*—That peculiar kind of sugar which is obtained from manna, and which gives the sweet flavor to celery and asparagus, has been lately converted by M. Sobrero into a highly fulminating compound. Although the process is not published, there is no doubt that, like fulminating cotton, the conversion is effected by the agency of sulphuric and nitric acids. It detonates by percussion as violently as fulminating mercury, and produces, during explosion, sufficient heat to ignite gunpowder. M. Sobrero has employed it as a substitute for fulminating mercury in percussion-caps. He found that a small quantity of mannite, crystallized from alcohol, and thus employed, discharged a gun as effectually as if fulminating mercury had been used. Further experiments are, however, required to show whether the mannite can be safely and economically substituted for mercury in the manufacture of percussion-caps.—*Ibid.*

